# Interpreting clinical outcomes using different strut thickness in coronary artery disease: insights from vascular imaging analysis

**DOI:** 10.3389/fcvm.2025.1491607

**Published:** 2025-03-04

**Authors:** Ju-Seung Kwun, Jin Joo Park, Si-Hyuck Kang, Sun-Hwa Kim, Chang-Hwan Yoon, Jung-Won Suh, Tae-Jin Youn, Kwang Soo Cha, Seung-Hwan Lee, Bum-Kee Hong, Seung-Woon Rha, Woong Chol Kang, In-Ho Chae

**Affiliations:** ^1^ Cardiovascular Center, Department of Internal Medicine, Seoul National University Bundang Hospital, Seongnam-si, Republic of Korea; ^2^ Cardiovascular Center, Department of Internal Medicine, Pusan National University Hospital, Busan, Republic of Korea; ^3^ Division of Cardiology, Department of Internal Medicine, Wonju Severance Hospital, Yonsei University College of Medicine, Seongnam-si, Republic of Korea; ^4^ Cardiovascular Center, Department of Internal Medicine, Gangnam Severance Hospital, Yonsei University College of Medicine, Seoul, Republic of Korea; ^5^ Cardiovascular Center, Department of Internal Medicine, Korea University Guro Hospital, Seoul, Republic of Korea; ^6^ Cardiovascular Center, Department of Internal Medicine, Gachon University Gil Medical Center, Incheon, Republic of Korea

**Keywords:** ultrathin-strut drug-eluting stent, quantitative coronary analysis, optical coherence tomography, percutaneous coronary intervention, coronary artery disease

## Abstract

**Background:**

Coronary artery disease is a global health concern that necessitates treatments, such as percutaneous coronary intervention (PCI) with drug-eluting stents (DES). Recent advancements in biodegradable polymer-coated DES have improved long-term outcomes by reducing neointimal hyperplasia. Superior long-term outcomes in patients with ultrathin-strut sirolimus-eluting Orsiro stent (BP-SES) compared with those with thick-strut biolimus-eluting BioMatrix stent (BP-BES) have been shown. This study aimed to explore the mechanisms underlying these differences by using quantitative coronary angiography (QCA) and optical coherence tomography (OCT).

**Methods:**

This sub-analysis of the BIODEGRADE trial, a prospective, randomized, multi-center study, compared BP-SES and BP-BES in patients who underwent PCI between July 2014 and September 2017. Patients with positive stress test results, ischemic symptoms, or those who consented to routine follow-up angiography were included. QCA and OCT were used to evaluate the lumen diameter, cross-sectional areas and stent apposition or coverage. OCT images were analyzed at 1 mm intervals within 5 mm proximal and distal to the stented segment.

**Results:**

Of the 2,341 patients, 689 underwent follow-up angiography between 18- and 36-months post-PCI, and 929 stents were analyzed via QCA. OCT images of 61 participants were available. The BP-SES group exhibited a significantly larger minimal lumen diameter and reduced late lumen loss compared to the BP-BES group (0.34 ± 0.45 mm vs. 0.42 ± 0.44 mm, *P* = 0.005). OCT analysis showed significantly less neointimal hyperplasia in the BP-SES group (0.04 ± 0.4 mm^2^ vs. 0.64 ± 0.54 mm^2^, *P* < 0.001), with no significant differences in stent strut coverage or inflammation markers, than in the BP-BES group.

**Conclusions:**

QCA and OCT analyses revealed less neointimal growth with BP-SES than with BP-BES, without delayed healing or increased inflammation. These findings underscore the importance of stent design characteristics and suggest that thinner struts may enhance clinical success by reducing restenosis and improving long-term vessel patency.

**Clinical Trial Registration:**

https://clinicaltrials.gov/study/NCT02299011 (NCT02299011).

## 1 Introduction

Coronary artery disease (CAD) remains a significant cause of morbidity and mortality worldwide and requires effective treatment, such as percutaneous coronary intervention (PCI) with drug-eluting stents (DES) (1, 2). The evolution of drug-eluting stent (DES) technology has enhanced long-term clinical outcomes by reducing neointimal hyperplasia (NIH), a key determinant of restenosis (3, 4). Among the various DES available, biodegradable polymer (BP)-coated DES, such as the thick-strut biolimus-eluting BioMatrix stent (BP-BES; Biosensors Inc., Newport Beach, CA) and ultrathin-strut sirolimus-eluting Orsiro stent (BP-SES; BIOTRONIK, Bulach, Switzerland) have been used as third-generation DES.

We previously reported a multicenter, randomized, open-label study comparing thick-strut BP-BES and ultrathin-strut BP-SES for CAD treatment, which provided valuable insights into the performance of these stents (5). The results of this study revealed that patients receiving ultrathin-strut BP-SES exhibited superior long-term clinical outcomes during the 36-month follow-up period than the thick strut BP-BES. This observation highlights the potential superiority of the ultrathin-strut thickness of the stent in retaining a larger vessel lumen area, similar to a previous report comparing stents with different strut thicknesses in the era of bare metal stents (6).

Given the compelling evidence suggesting a differential impact of these stents on the clinical outcomes associated with target lesion failure, further exploration and understanding of the underlying mechanisms are warranted. Therefore, this study aimed to investigate and elucidate the mechanisms contributing to the observed disparity between these stents by conducting quantitative coronary angiography (QCA) and optical coherence tomography (OCT) analyses in patients requiring PCI with DES.

## 2 Material and methods

### 2.1 Study design and participants

This study was a sub-analysis of the BIODEGRADE trial, a prospective, randomized, multi-center trial comparing the performance of the ultrathin-strut BP-SES and thick-strut BP-BES in patients who required PCI with DES implantation between July 2014 and September 2017 (5). We obtained angiography or OCT images in patients who had positive stress test results, experienced ischemic symptoms, or who consented to routine follow-up angiography between 18 and 36 months after the index PCI at the time of enrollment. QCA and OCT analyses were performed on these patients.

The study complied with the Declaration of Helsinki and was approved by the Institutional Review Board of Seoul National University Bundang Hospital (B-1403-244-002) and all participating centers. All patients provided written informed consent to participate in the trial and underwent imaging analysis using angiography or OCT before randomization.

### 2.2 QCA and OCT imaging and analysis

QCA examination using a conventional system (CAAS workstation 7.4, Pie Medical Imaging, The Netherlands) and OCT imaging assessments were performed using Dragonfly® image catheter and C7-XR™ OCT system (Abbott, USA) after an intra-coronary injection of nitroglycerine. From the OCT images, the cross-sectional area (CSA) of the coronary artery and stent strut apposition or coverage were evaluated using a dedicated imaging analysis program (OPTIS™ Imaging Systems software, Abbott Korea, Seoul, Korea). QCA and OCT image analysis was conducted by a single, experienced researcher from the core lab specializing in image analysis. The intraobserver variability of this researcher's analysis demonstrated an intraclass correlation coefficient (ICC) of over 0.9, indicating a very high level of reliability and also,this researcher has participated in numerous imaging studies previously conducted by our institution, ensuring reliable and consistent data (7, 8). Cross-sections at 1 mm intervals within 5 mm proximal and distal to the stented segment were analyzed. In each cross-section, lumen contours were drawn using a semi-automated detection algorithm and additional manual corrections when needed. For serial comparison, total stent length was measured post-procedure and on follow-up angiography; unchanged stent length was confirmed in all lesions. Inadequate images including noncircumferential CSA, poor quality or mismatched images, and cross sections with major side branches (diameter ≥2.0 mm) were excluded from the analysis.

In this analysis, neointimal thickness was measured as the perpendicular distance from the outer edge of the stent strut to the lumen surface, where a positive value indicated neointimal proliferation extending beyond the strut, reflecting endothelialization and tissue healing. Stent strut coverage was assessed by categorizing struts based on the neointimal thickness. Struts were considered fully covered embedded if the neointima completely enveloped the strut surface, covered protruded when more than 50% of the strut thickness was covered but not fully enveloped, uncovered when less than 50% of the strut thickness was covered with direct lumen contact, and malapposed if the struts were not in contact with the vessel wall, with a gap exceeding the expected strut thickness (Supplementary Figure S1). Coverage classification thresholds were applied based on stent type and size as follows: for Orsiro stents with ≤3.0 mm diameter (60 μm strut thickness), covered embedded struts were defined as those with a neointimal thickness ≥0 μm, covered protruded between −30 μm to 0 μm, uncovered between −60 μm to −30 *μ*m, and malapposed at <−60 μm; for Orsiro stents >3.0 mm (80 μm strut thickness), covered embedded struts had a neointimal thickness ≥0 μm, covered protruded between −40 μm to 0 μm, uncovered between −80 μm to −40 μm, and malapposed at <−80 μm; and for BioMatrix stents (120 μm strut thickness), covered embedded struts were defined at ≥0 μm, covered protruded between −60 μm to 0 μm, uncovered between −120 μm to −60 μm, and malapposed at <−120 μm. Thrombus was identified as a signal-rich mass with high backscattering and an irregular surface protruding into the lumen, indicating possible attachment to the stent strut or vessel wall and disrupting the smooth lumen contour. Peri-strut low-intensity area (PLIA) was defined as a low-intensity, homogeneous region surrounding the stent strut without signal attenuation on OCT, suggestive of inflammation or fibrin deposition, and was distinguished from lipid-rich plaques or necrotic cores by the absence of signal drop-out and its circumferential distribution around the strut.

### 2.3 Statistical analysis

The baseline characteristics of the study population were summarized as frequencies and percentages for categorical variables and as means and standard deviations for continuous variables. Continuous variables were compared between treatment groups using the two-sample *t*-test or Mann–Whitney *U*-test, depending on whether the data followed a normal distribution. The distributions of categorical variables were analyzed with the chi-squared (*χ*^2^) test. An empirical cumulative distribution function was estimated to represent the proportion of neointimal growth that was less than or equal to each value. To account for repeated measures within patients and potential covariates, generalized estimating equations was applied to determine the magnitude and statistical significance of the difference in neointimal growth between the two stent groups. This statistical method provides robust estimates by adjusting for intra-patient variability and covariates, ensuring accurate modeling of the relationships between variables (9). Statistical significance was set at a two-sided *P*-value of <0.05. Statistical analyses were performed using R version 3.1.0 (R Foundation for Statistical Computing, Vienna, Austria).

## 3 Results

### 3.1 Baseline characteristics

**Figure 1:**
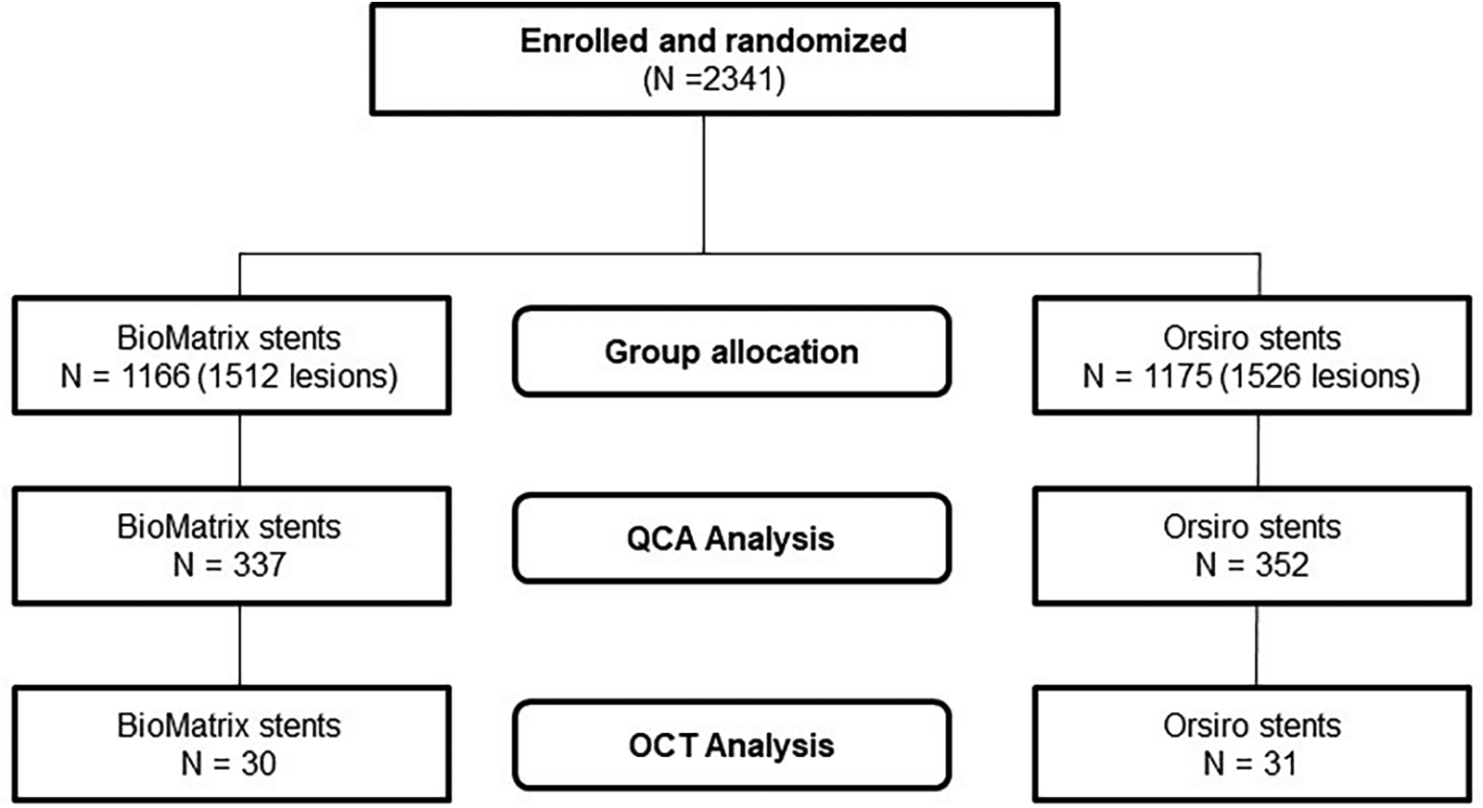
Flowchart representing the study design.

Among the 2,341 patients in the original study, 689 patients from six participating centers underwent angiography between 18 and 36 months after the index PCI, and 929 stents were analyzed for QCA (Figure 1). The OCT images of 61 participants from the two centers were available for analysis. Table 1 presents the baseline characteristics of the study population. No significant differences were observed between the two stent groups. However, there were significant differences in several characteristics between the groups that underwent follow-up angiography and those that did not (Supplementary Table S1). The group that underwent angiography had a lower mean age (62.55 ± 10.65 vs. 63.91 ± 10.96, *P* = 0.006), current smokers (24.2% vs. 28.3%, *P* < 0.001), and patients with atrial fibrillation (2.9% vs. 3.8%, *P* = 0.042), and a significantly higher prevalence of dyslipidemia (60.7% vs. 49.8%, *P* < 0.001) and a history of previous PCI (15.5% vs. 10.7%, *P* = 0.001). Regarding the clinical diagnoses, the angiography group had a higher percentage of patients with silent ischemia and stable angina, and a lower proportion of patients with unstable angina. For further analysis of the OCT group compared with the QCA group that did not undergo OCT, aside from the differences in the lower proportion of patients with ST-segment-elevation myocardial infarction in the OCT group, there were no significant differences in the baseline characteristics of patients (Supplementary Table S2).

**Table 1:** Baseline characteristics of the study population.

Variables	QCA analysis			OCT analysis		
	Total (*N* = 689)	Orsiro (*N* = 352)	BioMatrix (*N* = 337)	Total (*N* = 61)	Orsiro (*N* = 31)	BioMatrix (*N* = 30)
Age (years)	62.55 ± 10.65	62.47 ± 10.77	62.63 ± 10.54	61.18 ± 10.47	62.71 ± 10.54	59.60 ± 10.32
Men	514 (74.6)	254 (72.2)	260 (77.2)	43 (70.5)	21 (67.7)	22 (73.3)
Body mass index (kg/m^2^)	25.3 ± 3.23	25.29 ± 3.22	25.31 ± 3.24	25.34 ± 2.52	24.82 ± 2.77	25.87 ± 2.15
Diabetes mellitus	218 (31.6)	110 (31.2)	108 (32.0)	19 (31.1)	11 (35.5)	8 (26.7)
Arterial hypertension	416 (60.4)	209 (59.4)	207 (61.4)	39 (63.9)	24 (77.4)	15 (50.0)
Current smoker	167 (24.2)	84 (23.9)	83 (24.6)	14 (23.0)	7 (22.6)	7 (23.3)
Dyslipidemia	418 (60.7)	211 (59.9)	207 (61.4)	40 (65.6)	20 (64.5)	20 (66.7)
Previous percutaneous coronary intervention	107 (15.5)	54 (15.3)	53 (15.7)	10 (16.4)	8 (25.8)	2 (6.7)
Previous coronary artery bypass grafting	6 (0.9)	3 (0.9)	3 (0.9)	0 (0.0)	0 (0.0)	0 (0.0)
Previous myocardial infarction	44 (6.6)	26 (7.4)	18 (5.3)	5 (8.2)	4 (12.9)	1 (3.3)
Previous cerebrovascular accident	41 (6.0)	21 (6.0)	20 (5.9)	5 (8.2)	2 (6.5)	3 (10.0)
Atrial fibrillation	20 (2.9)	6 (1.7)	14 (4.2)	0 (0.0)	0 (0.0)	0 (0.0)
Clinical diagnosis for percutaneous coronary intervention						
Silent ischemia	56 (8.1)	23 (6.6)	33 (9.8)	2 (3.3)	0 (0.0)	2 (6.7)
Stable angina	221 (32.1)	113 (32.2)	108 (32.0)	22 (36.1)	11 (35.5)	11 (36.7)
Unstable angina	199 (28.9)	111 (31.6)	88 (26.1)	17 (27.9)	11 (35.5)	6 (20.0)
Non-ST-segment-elevation myocardial infarction	147 (21.4)	70 (19.9)	77 (22.8)	20 (32.8)	9 (29.0)	11 (36.7)
ST-segment-elevation myocardial infarction	65 (9.4)	34 (9.7)	31 (9.2)	0 (0.0)	0 (0.0)	0 (0.0)
Medication at discharge						
Aspirin	685 (99.4)	350 (99.4)	335 (99.4)	61 (100.0)	31 (100.0)	30 (100.0)
Clopidogrel	560 (81.3)	287 (81.5)	273 (81.0)	46 (75.4)	23 (74.2)	23 (76.7)
Ticagrelor	87 (12.6)	42 (11.9)	45 (13.4)	11 (18.0)	5 (16.1)	6 (20.0)
Prasugrel	36 (5.2)	20 (5.7)	16 (4.7)	4 (6.6)	3 (9.7)	1 (3.3)
Renin-angiotensin system inhibitors	203 (29.5)	97 (27.6)	106 (31.5)	12 (19.7)	4 (12.9)	8 (26.7)
Beta-blocker	435 (63.1)	220 (62.5)	215 (63.8)	35 (57.4)	20 (64.5)	15 (50.0)
Statin	657 (95.4)	340 (96.6)	317 (94.1)	60 (98.4)	30 (96.8)	30 (100.0)

Table 2 provides information on the characteristics of the target lesions and procedures. A total of 466 lesions treated with BP-SES and 463 lesions treated with BP-BES were included in the analysis. Except for a higher prevalence of chronic total occlusion lesions in the BP-SES group, no significant differences were observed between the two groups. When comparing the lesion characteristics analyzed by QCA to those of the group that did not undergo QCA, there were several significant differences (Supplementary Table S3). The proportions of lesion type C, chronic total occlusion, bifurcation lesions, and longer lesions were higher in the QCA group than in the non-QCA group. In addition, the QCA group had a higher number of stents per patient, greater total stent length per patient, lower proportion of cases in which direct stenting was used, and lower maximal pressure during the procedure. There were also several significant differences in the lesion characteristics between the OCT and non-OCT groups (Supplementary Table S4). Target vessels were located in the left main artery (11.8% vs. 4.2%, *P* = 0.007) and less located in the right coronary artery (11.8% vs. 29.0%, *P* = 0.002). The average stent diameter was longer, and a higher maximal pressure was applied in the OCT group.

**Table 2:** Lesion and procedure characteristics.

Variables	QCA analysis			OCT analysis		
	Total (*N* = 929)	Orsiro (*N* = 466)	BioMatrix (*N* = 463)	Total (*N* = 61)	Orsiro (*N* = 31)	BioMatrix (*N* = 30)
Target vessel location						
Left main artery (%)	45 (4.8)	19 (4.1)	26 (5.6)	6 (9.8)	4 (12.9)	2 (6.7)
Left anterior descending (%)	443 (47.7)	222 (47.6)	221 (47.7)	49 (80.3)	26 (83.9)	23 (76.7)
Left circumflex artery (%)	225 (24.2)	118 (25.3)	107 (23.1)	8 (13.1)	4 (12.9)	4 (13.3)
Right coronary artery (%)	256 (27.6)	124 (26.6)	132 (28.5)	5 (8.2)	2 (6.5)	3 (10.0)
Lesion type						
A	33 (3.6)	22 (4.7)	11 (2.4)	0 (0.0)	0 (0.0)	0 (0.0)
B1	221 (23.8)	111 (23.8)	110 (23.8)	14 (230)	7 (22.6)	7 (23.3)
B2	253 (27.2)	133 (28.5)	120 (25.9)	21 (34.4)	13 (41.9)	8 (26.7)
C	422 (45.4)	200 (42.9)	222 (47.9)	26 (42.6)	11 (35.5)	15 (50.0)
Chronic total occlusion	69 (7.4)	43 (9.2)	26 (5.6)	3 (4.9)	1 (3.2)	2 (6.7)
Bifurcation lesion	166 (17.9)	74 (15.9)	92 (19.9)	13 (21.3)	8 (25.8)	5 (16.7)
Lesion length (mm)	22.33 ± 10.66	22.27 ± 10.90	22.40 ± 10.42	24.79 ± 12.88	25.69 ± 14.64	23.87 ± 10.94
Direct stenting	52 (5.6)	25 (5.4)	27 (5.8)	3 (4.9)	2 (6.5)	1 (3.3)
Number of stents per patient	1.53 ± 0.77	1.51 ± 0.78	1.54 ± 0.76	1.16 ± 0.45	1.19 ± 0.54	1.13 ± 0.35
Number of stents per lesion	1.13 ± 0.38	1.14 ± 0.39	1.12 ± 0.38	1.16 ± 0.45	1.19 ± 0.54	1.13 ± 0.35
Total stent length per patient lesion	36.89 ± 22.00	36.07 ± 22.35	37.73 ± 21.62	29.89 ± 14.72	30.94 ± 15.60	28.80 ± 13.94
Total stent length per lesion	27.36 ± 13.24	27.25 ± 13.33	27.46 ± 13.17	29.89 ± 14.72	30.94 ± 15.60	28.80 ± 13.94
Sum of stent length per lesion (mm)						
<35	734 (79.0)	364 (78.1)	370 (79.9)	45 (73.8)	23 (74.2)	22 (73.3)
≥35	195 (21.0)	102 (21.9)	93 (20.1)	16 (26.2)	8 (25.8)	8 (26.7)
Average stent diameter (mm)	3.01 ± 0.43	3.01 ± 0.44	3.00 ± 0.43	3.18 ± 0.39	3.20 ± 0.39	3.17 ± 0.40
Minimum stent diameter per lesion (mm)						
<3	391 (42.1)	196 (42.1)	195 (42.1)	13 (21.3)	6 (19.4)	7 (23.3)
≥3	538 (57.9)	270 (57.9)	268 (57.9)	48 (78.7)	25 (80.6)	23 (76.7)
Maximal pressure (atm)	9.72 ± 3.23	10.36 ± 3.07	9.07 ± 3.27	10.52 ± 3.98	11.19 ± 3.94	9.83 ± 3.97
Acute gain (mm)	2.07 ± 0.6	2.10 ± 0.61	2.04 ± 0.60	2.15 ± 0.58	2.18 ± 0.60	2.13 ± 0.56

### 3.2 Intracoronary artery image analysis

**Figure 2:**
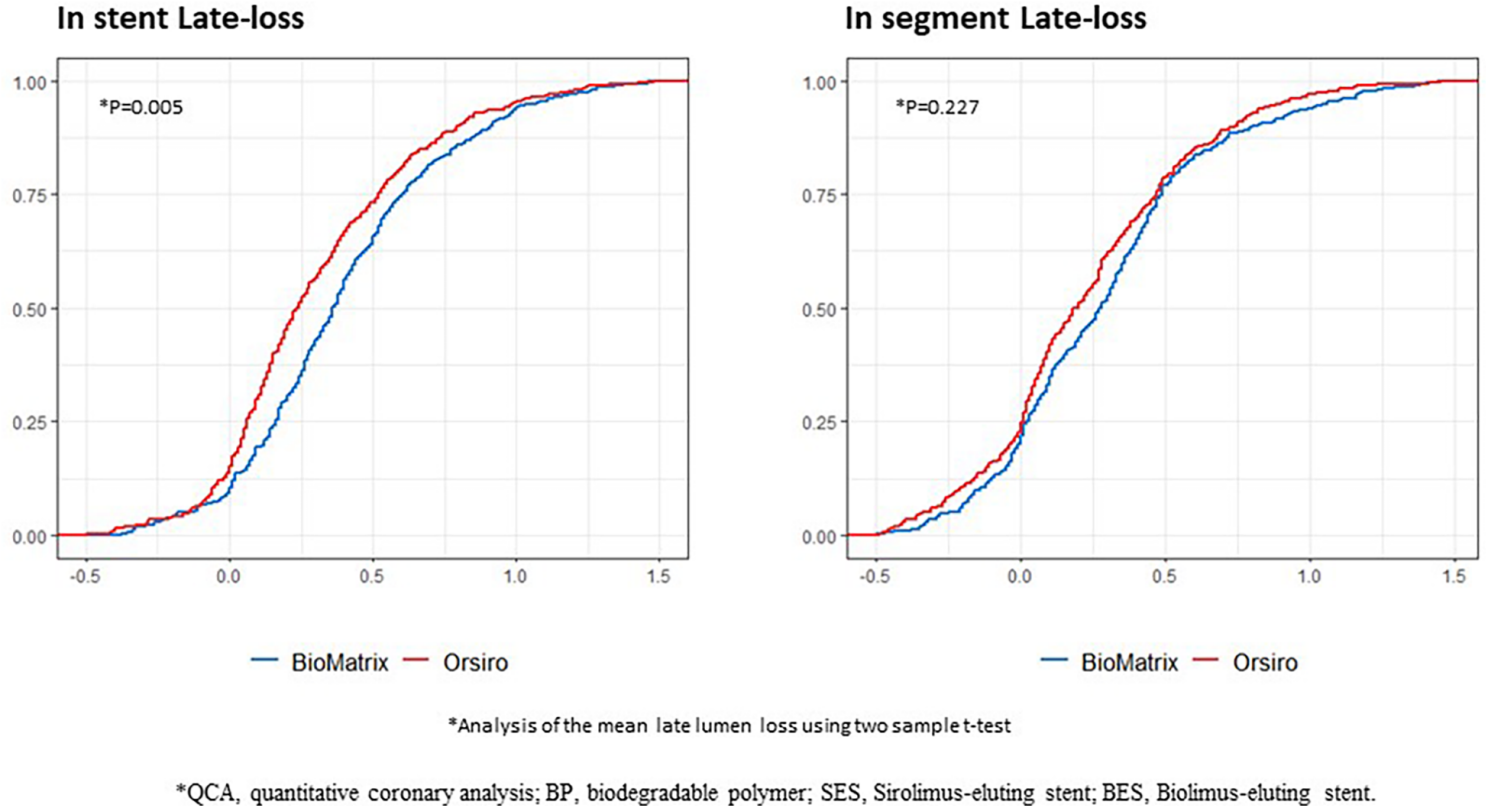
Cumulative probability distribution of the QCA results between the BP-SES and BP-BES. QCA, quantitative coronary analysis; BP, biodegradable polymer; SES, Sirolimus-eluting stent; BES, Biolimus-eluting stent; *X*-axis represents the area (mm^2^), and the *Y*-axis represents the rate.

Table 3 summarizes representative follow-up QCA and OCT data. There was no difference in the minimal lumen diameter between the two groups post-PCI; however, BP-SES showed a significantly larger minimal lumen diameter than BP-BES at follow-up. As a result, the BP-SES exhibited significantly reduced late lumen loss compared to the BP-BES (0.34 ± 0.45 mm vs. 0.42 ± 0.44 mm, *P* = 0.005). The cumulative probability distribution of in-stent late lumen loss measured using QCA clearly showed a lower late lumen loss in the BP-SES group than in the BP-BES group (Figure 2). However, no statistically significant difference in the in-segment late lumen loss was observed between the two stent types (Table 3 and Figure 2).

**Table 3:** OCT and QCA lesion analysis.

Variables	Post	Follow-up	Post	Follow-up	Post	Follow-up
QCA	Orsiro (*N* = 466)		BioMatrix (*N* = 463)		*P*	*P*
In Stent						
Minimal lumen diameter (mm)	2.69 ± 0.56	2.34 ± 0.60	2.63 ± 0.56	2.21 ± 0.62	0.156	0.001
Late loss (mm)	–	0.34 ± 0.45	12.59 ± 8.27	0.42 ± 0.44	0.811	0.005
Diameter stenosis (%)	12.72 ± 8.05	17.77 ± 14.2	2.38 ± 0.61	20.66 ± 15.1	0.211	0.003
In Segment						
Minimal lumen diameter (mm)	2.43 ± 0.60	2.18 ± 0.60	20.36 ± 9.07	2.09 ± 0.59	0.423	0.027
Late loss (mm)	–	0.25 ± 0.47		0.29 ± 0.44		0.227
Diameter stenosis (%)	19.90 ± 8.39	22.95 ± 13.95		23.91 ± 13.87		0.294
OCT	Orsiro (*N* = 31)		BioMatrix (*N* = 30)		*P*	*P*
Mean lumen CSA (mm^2^)	8.04 ± 2.25	7.86 ± 2.24	7.88 ± 2.04	6.75 ± 2.5	0.765	0.072
Mean stent CSA (mm^2^)	7.63 ± 2.14	7.9 ± 2.2	7.33 ± 1.97	7.39 ± 2.34	0.566	0.38
Mean NIH CSA (mm^2^)		0.04 ± 0.4		0.64 ± 0.54		<.001
Covered embedded (%)		89.78 ± 16.16		94.83 ± 8.67		0.106
Covered protruded (%)		4.32 ± 7.17		3.78 ± 6.50		0.360
Uncovered (%)		3.07 ± 5.95		1.00 ± 2.01		0.056
Malapposed (%)	12.69 ± 8.60	2.83 ± 3.96	6.55 ± 7.27	0.39 ± 1.01	<.001	<.001

Values are presented as *n* (%) or mean ± standard deviation.

OCT, optical coherence tomography; QCA, quantitative coronary analysis; CSA, cross-sectional area; NIH, neointimal hyperplasia.

**Figure 3:**
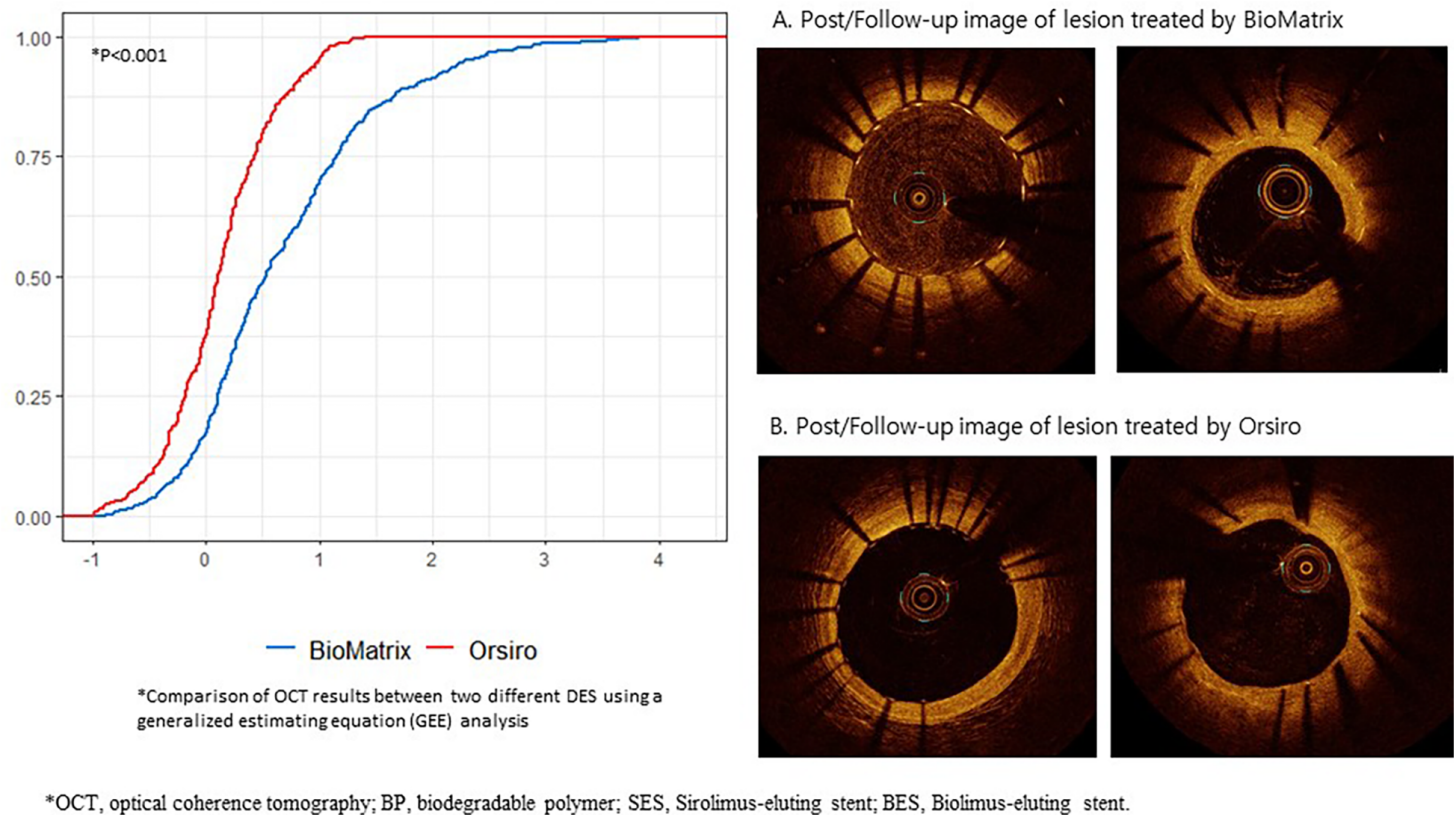
Cumulative probability distribution of the OCT results between the BP-SES and BP-BES, and their representative cases of neointimal change. OCT, optical coherence tomography; BP, biodegradable polymer; SES, Sirolimus-eluting stent; BES, Biolimus-eluting stent; *X*-axis represents the area (mm^2^), and the *Y*-axis represents the rate.

In the OCT analysis, the BP-SES group tended to have a larger mean lumen area (7.86 ± 2.24 mm^2^ vs. 6.75 ± 2.50 mm^2^, *P* = 0.072), and significantly lower mean NIH than those in the thick-strut BP-BES group (0.04 ± 0.4 mm^2^ vs. 0.64 ± 0.54 mm^2^, *P* < 0.001). Figure 3 clearly illustrates the cumulative probability distribution graph showing a lower mean NIH with the BP-SES than with the BP-BES; the estimated difference between the two stent types was −0.568 mm^2^ (*P* < 0.001), indicating a statistically significant decrease in NIH with the ultrathin-strut stent. Furthermore, per-cross section analysis revealed that the mean NIH CSA was also significantly lower in the BP-SES group compared to the BP-BES group (0.04 ± 0.4 mm^2^ vs. 0.64 ± 0.54 mm^2^, *P* < 0.001) (Supplementary Table S5). Representative cases demonstrating changes in the neointimal coverage are shown in Figure 3.

The analysis of strut apposition and coverage revealed that the proportion of malapposed struts was significantly higher in the BP-SES group at post-PCI and follow-up (12.69 ± 19.2% and 2.75 ± 8.64%) than the BP-BES group (6.55 ± 7.27% vs. 0.39 ± 1.01%) (*P* < 0.001, respectively) (Table 3). In addition, this trend persisted when analyzed at both the per-cross section and per-strut levels (Supplementary Tables S5 and S6). However, the proportion of covered embedded, covered protruded and uncovered struts was not significantly different between the BP-SES and the BP-SES group at follow-up (Table 3). At the per-cross section and per-strut level, the BP-SES group had a significantly lower number of covered embedded struts and higher uncovered struts compared to the BP-BES group (Supplementary Tables S5 and S6). We also reviewed the OCT images to compare the two stent groups in terms of subclinical stent thrombosis, presence of macrophages, and PLIA. There were no cases of PLIA, or subclinical stent thrombosis observed in either group, indicating no significant differences in delayed healing or inflammation after stenting.

### 3.3 Clinical outcomes

When comparing ultrathin BP-SES to thick BP-BES in the angiography follow-up group, there were no significant differences in the clinical outcomes (Table 4). However, when comparing the angiography follow-up group with the group that did not undergo follow-up (Supplementary Table S5), there was a significantly lower proportion of deaths, including all-cause, cardiac, and non-cardiac deaths. Any myocardial infarction, ischemia-driven target lesion/vessel revascularization, or any repeat revascularization was higher in the angiography follow-up group. No target lesion failure was observed in the OCT group (Supplementary Table S6).

**Table 4:** Clinical outcomes.

Variables	Orsiro (*N* = 352)	BioMatrix (*N* = 337)	*P* (*χ*2 test)	Hazard ratio (95% confidence interval)	*P*
Target lesion failure	11 (3.1)	17 (5.0)	0.279	0.61 (0.29–1.31)	0.207
Death					
All-cause death	6 (1.7)	3 (0.9)	0.506	1.92 (0.48–7.66)	0.357
Cardiac death	1 (1.1)	2 (0.6)	0.687	1.92 (0.35–10.46)	0.453
Noncardiac death	2 (0.6)	1 (0.3)	>0.999	1.92 (0.17–21.15)	0.595
Target vessel-related myocardial infarction	2 (0.6)	1 (0.3)	>0.999	1.92 (0.17–21.17)	0.594
Any myocardial infarction	7 (2.0)	2 (0.6)	0.178	3.39 (0.70–16.31)	0.128
Ischemia-driven target lesion revascularization	8 (2.3)	14 (4.2)	0.235	0.54 (0.23–1.29)	0.168
Ischemia-driven target vessel revascularization	14 (4.0)	24 (7.1)	0.101	0.55 (0.29–1.07)	0.080
Any repeat revascularization	28 (8.0)	38 (11.3)	0.177	0.70 (0.43–1.15)	0.158
Stent thrombosis^a^	1 (0.3)	0 (0.0)	>0.999		
Patient-oriented composite endpoint	34 (9.7)	40 (11.9)	0.416	0.82 (0.52–1.29)	0.383
Bleeding	11 (3.1)	8 (2.4)	0.712	1.33 (0.53–3.30)	0.544

^a^ Stent thrombosis: one definite subacute stent thrombosis leading to myocardial infarction; two definite late stent thromboses causing unstable angina and myocardial infarction; one definite very late stent thrombosis leading to myocardial infarction.

## 4 Discussion

In this study, we aimed to investigate the intravascular characteristics of thick-strut BP-BES and ultrathin-strut BP-SES to gain insights into the differences in clinical outcomes between the two groups. BP-SES showed less neointimal growth than BP-BES, and there was no evidence of delayed healing or inflammation in either group.

Previous studies have demonstrated that ultrathin-strut DES exhibit superior clinical outcomes (5, 10, 11). However, the specific reason for this remains unclear. To address this gap, we performed QCA and intravascular imaging using OCT to obtain detailed insights into the underlying mechanisms contributing to these differences.

Our findings contribute to the understanding of the clinical superiority of ultrathin-strut BP-SES over thick-strut BP-BES. QCA allowed us to examine the differences in lumen narrowing caused by different strut thicknesses, and the OCT images further explored the nature of neointimal growth and vessel healing characteristics in a comprehensive manner. We observed significantly less NIH in the ultrathin-strut BP-SES group but no difference in vascular healing characteristics in terms of subclinical stent thrombosis, presence of macrophages, or peri-strut low-intensity area. These findings align with previous studies demonstrating the benefits of the ultrathin-strut BP-SES in reducing inflammation and neointimal growth while maintaining favorable vascular healing characteristics (12–14). Moreover, the stent strut malapposition was observed more frequently in the BP-SES group. Although previous studies have reported an association between malapposition and stent thrombosis in the first-generation DES (15, 16), no differences in clinical outcomes were observed with the ultrathin-strut Orsiro stent in this study.

To the best of our knowledge, no previous studies have presented detailed comparisons between ultrathin-strut BP-SES and thick-strut BP-BES using intravascular imaging. Our study fills this gap by providing a comprehensive analysis and quantification of the NIH. The reduced NIH observed in the ultrathin-strut group can be attributed to several factors related to its design characteristics. The unique design of Orsiro BP-SES, which combines an ultrathin cobalts-chromium platform (60 μm for ≤3.0 mm stents or 80 μm for >3.0 mm stents) with a thin passive coating of amorphous silicon carbide may reduce the disturbance of blood flow and improve endothelization (17). In addition, it contributes to minimal endothelial damage and reduces long-term inflammation at the vessel interface (18).

Our findings have several significant clinical implications. The lower neointimal growth in BP-DES suggests a potentially reduced risk of restenosis and target lesion revascularization without an increased risk of stent thrombosis (19, 20). These improved target lesion-related outcomes have important implications for long-term clinical success and patient prognosis; hence, this study has significant implications for the future development of DES, highlighting the importance of design characteristics in stent performance, wherein the thinner the stent, the better.

### 4.1 Limitations

Despite the limitations posed by the high cost and invasive nature of OCT, enrolling a large number of patients in this study was challenging. Performing invasive angiography in asymptomatic patients without evident clinical indications revealed further difficulties. Although the sample size was relatively small, this study is valuable because it incorporates OCT analysis, which was previously lacking in comparative studies of thick-strut vs. ultrathin-strut DES. The inclusion of QCA and OCT provides a unique perspective on the mechanisms underlying the observed differences in clinical outcomes. The scarcity of previous publications that incorporated detailed comparisons using QCA and OCT strengthened the significance of this study. In addition, there were some differences in the baseline and lesion characteristics, and several clinical outcomes between the selected angiography follow-up group and the group that did not undergo follow-up. Overall, angiography follow-up was more frequent in event-free patients. Thus, conducting an analysis between these groups could have potentially led to an underestimated result. Because the majority of follow-up angiography was conducted in agreed-upon patients, randomization was difficult to implement. In addition, the nature of the follow-up analysis, which focused on the surviving patients, inevitably led to these results.

Moreover, the selection process for follow-up examinations introduces a high probability of selection bias. Patients who consented to follow-up angiography may inherently differ in clinical or demographic characteristics from those who did not, potentially influencing the study outcomes. This selection bias could limit the generalizability of the findings and lead to an underestimation or overestimation of adverse events or clinical benefits.

Despite these challenges, it is important to note that the comparison of lesion characteristics between the stent groups was relatively balanced, even with the inclusion of patients with more anatomical challenges in the follow-up group. This suggests that the results were not biased towards a more favorable outcome for the follow-up angiography group, and the comparison was conducted as fairly as possible, given the limitations. To address these potential issues, a randomized approach with a larger sample size would be beneficial in future studies.

### 4.2 Conclusion

QCA and OCT showed less neointimal growth in ultrathin-strut BP-SES than in thick-strut BP-BES without any evidence of delayed healing or inflammation. These results provide insights into the clinical outcomes of BP-SES.

## Data Availability

The raw data supporting the conclusions of this article will be made available by the authors, without undue reservation.
