# Supplementary figures and images for: Interpreting clinical outcomes using different strut thickness in coronary artery disease: insights from vascular imaging analysis
Source: Front Cardiovasc Med. 2025 Mar 4;12:1491607. doi: 10.3389/fcvm.2025.1491607 (PMC11913801; doi:10.3389/fcvm.2025.1491607)

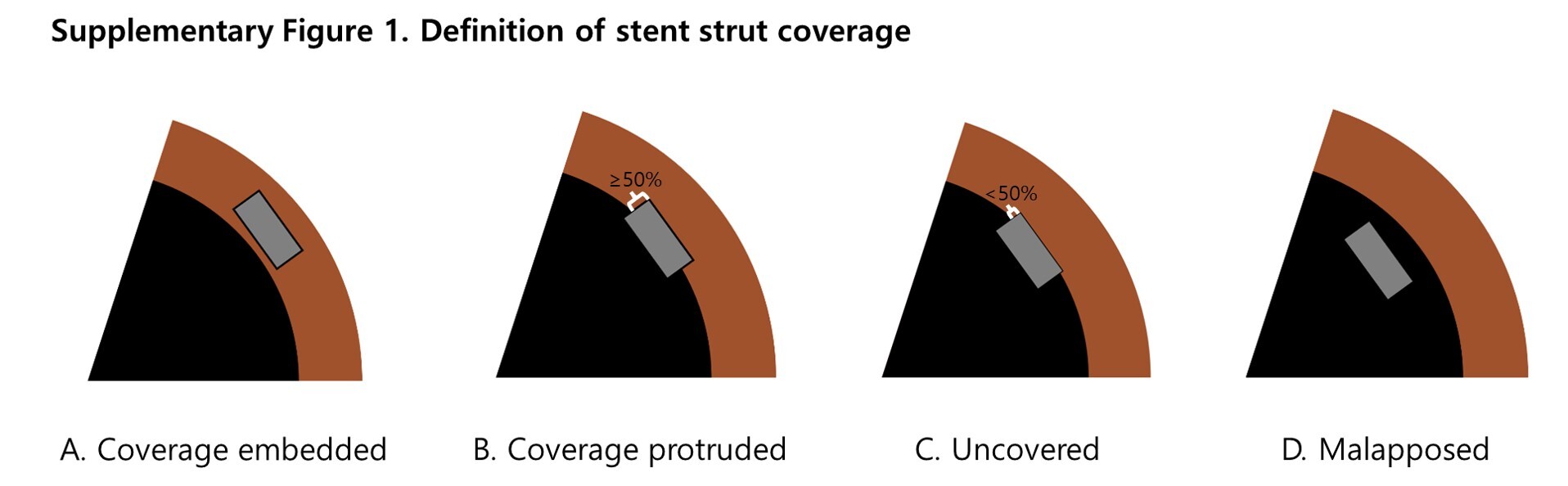

Supplement: Supplementary file 1 [file Image1.jpeg]
