# Supplementary material for: Interpreting clinical outcomes using different strut thickness in coronary artery disease: insights from vascular imaging analysis
Source: Front Cardiovasc Med. 2025 Mar 4;12:1491607. doi: 10.3389/fcvm.2025.1491607 (PMC11913801; doi:10.3389/fcvm.2025.1491607)
Supplement: Supplementary file 2 [file Table1.docx]

Supplementary Material

# Supplementary Tables

**Supplementary Table 1.** Comparison of baseline characteristics between patients who underwent angiography and those who did not.

|  | **Total**  **(N=2327)** | **YES**  **(N=689)** | **NO**  **(N=1638)** | ***P*** |
| --- | --- | --- | --- | --- |
| Age (years) | 63.51±10.88 | 62.55±10.65 | 63.91±10.96 | 0.006 |
| Men | 1673 (71.9) | 514 (74.6) | 1159 (70.8) | 0.067 |
| Body mass index (kg/m^2^) | 25.09±3.35 | 25.3±3.23 | 25.0±3.40 | 0.053 |
| Diabetes mellitus | 777 (33.4) | 218 (31.6) | 559 (34.1) | 0.266 |
| Arterial hypertension | 1391 (59.8) | 416 (60.4) | 975 (59.5) | 0.736 |
| Current smoker | 630 (27.1) | 167 (24.2) | 463 (28.3) | <.001 |
| Dyslipidemia | 1234 (53) | 418 (60.7) | 816 (49.8) | <.001 |
| Previous percutaneous coronary intervention | 282 (12.1) | 107 (15.5) | 175 (10.7) | 0.001 |
| Previous coronary artery bypass grafting | 18 (0.8) | 6 (0.9) | 12 (0.7) | 0.93 |
| Previous myocardial infarction | 116 (5) | 44 (6.6) | 72 (4.4) | 0.056 |
| Previous cerebrovascular accident | 161 (6.9) | 41 (6.0) | 120 (7.3) | 0.268 |
| Atrial fibrillation | 82 (3.5) | 20 (2.9) | 62 (3.8) | 0.042 |
| Clinical diagnosis for percutaneous coronary intervention |  |  |  | <.001 |
| Silent ischemia | 120 (5.2) | 56 (8.1) | 64 (3.9) |  |
| Stable angina | 641 (27.6) | 221 (32.1) | 420 (25.6) |  |
| Unstable angina | 848 (36.5) | 199 (28.9) | 649 (39.6) |  |
| Non-ST-segment-elevation myocardial infarction | 495 (21.3) | 147 (21.4) | 348 (21.2) |  |
| ST-segment-elevation myocardial infarction | 222 (9.5) | 65 (9.4) | 157 (9.6) |  |
| Medication at discharge |  |  |  |  |
| Aspirin | 2302 (99) | 685 (99.4) | 1617 (98.8) | 0.241 |
| Clopidogrel | 1922 (82.6) | 560 (81.3) | 1362 (83.2) | 0.29 |
| Ticagrelor | 292 (12.6) | 87 (12.6) | 205 (12.5) | 0.999 |
| Prasugrel | 82 (3.5) | 36 (5.2) | 46 (2.8) | 0.006 |
| Renin-angiotensin system inhibitors | 720 (31) | 203 (29.5) | 517 (31.6) | 0.343 |
| Beta-blocker | 1464 (63) | 435 (63.1) | 1029 (62.9) | 0.951 |
| Statin | 2195 (94.4) | 657 (95.4) | 1538 (94.0) | 0.214 |

**Supplementary Table 2.** Comparison of baseline characteristics between patients who underwent OCT analysis and those who underwent angiography only.

|  | **Total**  **(N=689)** | **OCT**  **(N=61)** | **Non-OCT**  **(N=628)** | ***P*** |
| --- | --- | --- | --- | --- |
| Age (years) | 62.55±10.65 | 61.18±10.47 | 62.68±10.66 | 0.293 |
| Men | 514 (74.6) | 43 (70.5) | 471 (75.0) | 0.536 |
| Body mass index (kg/m^2^) | 25.3±3.23 | 25.34±2.52 | 25.29±3.29 | 0.905 |
| Diabetes mellitus | 218 (31.6) | 19 (31.1) | 199 (31.7) | >.999 |
| Arterial hypertension | 416 (60.4) | 39 (63.9) | 377 (60.0) | 0.647 |
| Current smoker | 167 (24.2) | 14 (23.0) | 153 (24.4) | 0.092 |
| Dyslipidemia | 418 (60.7) | 40 (65.6) | 378 (60.2) | 0.494 |
| Previous percutaneous coronary intervention | 107 (15.5) | 10 (16.4) | 97 (15.4) | 0.992 |
| Previous coronary artery bypass grafting | 6 (0.9) | 0 (0.0) | 6 (1.0) | 0.964 |
| Previous myocardial infarction | 44 (6.6) | 5 (8.2) | 39 (6.2) | 0.740 |
| Previous cerebrovascular accident | 41 (6.0) | 5 (8.2) | 36 (5.7) | 0.622 |
| Atrial fibrillation | 20 (2.9) | 0 (0.0) | 20 (3.2) | 0.218 |
| Clinical diagnosis for percutaneous coronary intervention |  |  |  | 0.013 |
| Silent ischemia | 56 (8.1) | 2 (3.3) | 54 (8.6) |  |
| Stable angina | 221 (32.1) | 22 (36.1) | 199 (31.7) |  |
| Unstable angina | 199 (28.9) | 17 (27.9) | 182 (29.0) |  |
| Non-ST-segment-elevation myocardial infarction | 147 (21.4) | 20 (32.8) | 127 (20.3) |  |
| ST-segment-elevation myocardial infarction | 65 (9.4) | 0 (0.0) | 65 (10.4) |  |
| Medication at discharge |  |  |  |  |
| Aspirin | 685 (99.4) | 61 (100.0) | 624 (99.4) | >.999 |
| Clopidogrel | 560 (81.3) | 46 (75.4) | 514 (81.8) | 0.290 |
| Ticagrelor | 87 (12.6) | 11 (18.0) | 76 (12.1) | 0.259 |
| Prasugrel | 36 (5.2) | 4 (6.6) | 32 (5.1) | 0.850 |
| Renin-angiotensin system inhibitors | 203 (29.5) | 12 (19.7) | 191 (30.5) | 0.106 |
| Beta-blocker | 435 (63.1) | 35 (57.4) | 400 (63.7) | 0.402 |
| Statin | 657 (95.4) | 60 (98.4) | 597 (95.1) | 0.396 |

**Supplementary Table 3.** Comparison of lesion characteristics between stents with follow-up angiography and those without follow-up angiography.

|  | **Total**  **(N=3022)** | **YES**  **(N=929)** | **NO**  **(N=2093)** | ***P*** |
| --- | --- | --- | --- | --- |
| Target vessel location |  |  |  |  |
| Left main artery (%) | 100 (3.3) | 45 (4.8) | 55 (2.6) | 0.348 |
| Left anterior descending (%) | 1503 (49.7) | 443 (47.7) | 1060 (50.6) | 0.144 |
| Left circumflex artery (%) | 684 (22.6) | 225 (24.2) | 459 (21.9) | 0.18 |
| Right coronary artery (%) | 825 (27.3) | 256 (27.6) | 569 (27.2) | 0.868 |
| Lesion type |  |  |  | <.001 |
| A | 222 (7.3) | 33 (3.6) | 189 (9.0) |  |
| B1 | 805 (26.6) | 221 (23.8) | 584 (27.9) |  |
| B2 | 773 (25.6) | 253 (27.2) | 520 (24.8) |  |
| C | 1222 (40.4) | 422 (45.4) | 800 (38.2) |  |
| Chronic total occlusion | 164 (5.4) | 69 (7.4) | 95 (4.5) |  |
| Bifurcation lesion | 449 (14.9) | 166 (17.9) | 283 (13.5) | 0.002 |
| Lesion length (mm) | 21.78_10.13 | 22.33±10.66 | 21.53_9.87 | 0.05 |
| Direct stenting | 346 (11.4) | 52 (5.6) | 294 (14.0) | <.001 |
| Number of stents per patient | 1.45_0.75 | 1.55±0.83 | 1.41_0.70 | <.001 |
| Number of stents per lesion | 1.12_0.35 | 1.13±0.38 | 1.11_0.34 | 0.228 |
| Total stent length per patient lesion | 34.94_21.25 | 37.59±23.22 | 33.82_20.26 | <.001 |
| Total stent length per lesion | 26.9_12.46 | 27.36±13.24 | 26.70_12.09 | 0.196 |
| Sum of stent length per lesion (mm) |  |  |  | 0.493 |
| <35 | 2412 (79.8) | 734 (79.0) | 1678 (80.2) |  |
| ≥35 | 610 (20.2) | 195 (21.0) | 415 (19.8) |  |
| Average stent diameter (mm) | 3.02_0.42 | 3.01±0.43 | 3.03_0.42 | 0.238 |
| Minimum stent diameter per lesion (mm) |  |  |  | 0.877 |
| <3 | 1264 (41.8) | 391 (42.1) | 873 (41.7) |  |
| ≥3 | 1758 (58.2) | 538 (57.9) | 1220 (58.3) |  |
| Maximal pressure (atm) | 10.72_3.64 | 9.72±3.23 | 11.16_3.72 | <.001 |
| Acute gain (mm) | 2.08_0.61 | 2.07±0.6 | 2.09_0.62 | 0.298 |

**Supplementary Table 4.** Comparison of lesion characteristics between stents with OCT analysis and those with follow-up angiography only.

|  | **Total**  **(N=929)** | **OCT**  **(N=76)** | **Non-OCT**  **(N=853)** | ***P*** |
| --- | --- | --- | --- | --- |
| Target vessel location |  |  |  |  |
| Left main artery (%) | 45 (4.8) | 9 (11.8) | 36 (4.2) | 0.007 |
| Left anterior descending (%) | 443 (47.7) | 55 (72.4) | 388 (45.5) | >.999 |
| Left circumflex artery (%) | 225 (24.2) | 12 (15.8) | 213 (25.0) | 0.099 |
| Right coronary artery (%) | 256 (27.6) | 9 (11.8) | 247 (29.0) | 0.002 |
| Lesion type |  |  |  | 0.908 |
| A | 33 (3.6) | 2 (2.6) | 31 (3.6) |  |
| B1 | 221 (23.8) | 18 (23.7) | 203 (23.8) |  |
| B2 | 253 (27.2) | 23 (30.3) | 230 (27.0) |  |
| C | 422 (45.4) | 33 (43.4) | 389 (45.6) |  |
| Chronic total occlusion | 69 (7.4) | 3 (3.9) | 66 (7.7) | 0.328 |
| Bifurcation lesion | 166 (17.9) | 16 (21.1) | 150 (17.6) | 0.549 |
| Lesion length (mm) | 22.33±10.66 | 24.79±12.39 | 22.12±10.47 | 0.072 |
| Direct stenting | 52 (5.6) | 6 (7.9) | 46 (5.4) | 0.516 |
| Number of stents per patient | 1.53±0.77 | 1.43±0.74 | 1.54±0.77 | 0.294 |
| Number of stents per lesion | 1.13±0.38 | 1.14±0.42 | 1.13±0.38 | 0.750 |
| Total stent length per patient lesion | 36.89±22.00 | 36.80±22.33 | 36.89±21.98 | 0.976 |
| Total stent length per lesion | 27.36±13.24 | 29.54±14.13 | 27.16±13.15 | 0.134 |
| Sum of stent length per lesion (mm) |  |  |  | 0.103 |
| <35 | 734 (79.0) | 54 (71.1) | 680 (79.7) |  |
| ≥35 | 195 (21.0) | 22 (28.9) | 173 (20.3) |  |
| Average stent diameter (mm) | 3.01±0.43 | 3.17±0.41 | 2.99±0.43 | 0.001 |
| Minimum stent diameter per lesion (mm) |  |  |  | 0.001 |
| <3 | 391 (42.1) | 18 (23.7) | 373 (43.7) |  |
| ≥3 | 538 (57.9) | 58 (76.3) | 480 (56.3) |  |
| Maximal pressure (atm) | 9.72±3.23 | 10.70±4.01 | 9.63±3.14 | 0.027 |
| Acute gain (mm) | 2.07±0.6 | 2.14±0.59 | 2.06±0.61 | 0.286 |

**Supplementary Table 5.** OCT lesion analysis per cross-section

| **OCT** | **Orsiro (N=1,777)** | | **BioMatrix (N=1,534)** | | ***P*** | ***P*** |
| --- | --- | --- | --- | --- | --- | --- |
|  | **Post** | **Follow-up** | **Post** | **Follow-up** |  |  |
| Mean lumen CSA (mm^2^) | 7.77±2.63 7.57±2.46 | | 7.73±2.39 6.50±2.66 | | 0.935 | <.001 |
| Mean stent CSA (mm^2^) | 7.38±2.47 7.61±2.37 | | 7.18±2.20 7.17±2.42 | | 0.130 | <.001 |
| Mean NIH CSA (mm^2^) | 0.04±0.64 | |  | 0.67±0.88 |  | <.001 |
| Covered embedded (%) | 90.3±22.90 | |  | 94.60±16.60 |  | <.001 |
| Covered protruded (%) | 4.05±11.90 | |  | 4.02±13.40 |  | 0.151 |
| Uncovered (%) | 2.86±8.96 | |  | 1.06±4.74 |  | <.001 |
| Malapposed (%) | 12.69±19.2 2.75±8.64 | | 6.34±14.2 | 0.27±2.39 | <.001 | <.001 |

**Supplementary Table 6.** OCT lesion analysis per strut

| **OCT** | **Orsiro (N=9,595)** | **Orsiro (N=9,595)** | **BioMatrix (N=7,690)** | **BioMatrix (N=7,006)** | ***P*** | ***P*** |
| --- | --- | --- | --- | --- | --- | --- |
|  | **Post** | **Follow-up** | **Post** | **Follow-up** |  |  |
| Covered embedded struts (%) |  | 8788 (91.5) |  | 6641 (94.8) |  | <.001 |
| Covered protruded struts (%) |  | 348 (3.6%) |  | 276 (3.9) |  | 0.302 |
| Uncovered struts (%) |  | 237 (2.5) |  | 71 (1.0) |  | <.001 |
| Malapposed struts (%) | 1302 (13.6) | 234 (2.4) | 454 (5.9) | 18 (0.3) | <.001 | <.001 |

**Supplementary Table 7.** Clinical outcome of patients with angiography follow-up and patients without angiography follow-up

|  | **Yes**  **(N=689)** | **No**  **(N=1,638)** | ***P* (Chi-squared test)** | **Hazard ratio (95% confidence interval)** | ***P*** |
| --- | --- | --- | --- | --- | --- |
| Target lesion failure | 28 (4.1) | 68 (4.2) | >.999 | 0.96 (0.62-1.49) | 0.856 |
| Death |  |  |  |  |  |
| All-cause death | 9 (1.3) | 86 (5.3) | <.001 | 0.24 (0.12-0.48) | <.001 |
| Cardiac death | 6 (0.9) | 41 (2.5) | 0.017 | 0.34 (0.14-0.80) | 0.014 |
| Noncardiac death | 3 (0.4) | 45 (2.7) | 0.001 | 0.15 (0.05-0.50) | 0.002 |
| Target vessel-related myocardial infarction | 3 (0.4) | 4 (0.2) | 0.429 | 1.75 (0.39-7.80) | 0.466 |
| Any myocardial infarction | 9 (1.3) | 7 (0.4) | 0.027 | 3.01 (1.12-8.09) | 0.029 |
| Ischemia-driven target lesion revascularization | 22 (3.2) | 29 (1.8) | 0.047 | 1.77 (1.01-3.07) | 0.044 |
| Ischemia-driven target vessel revascularization | 38 (5.5) | 43 (2.6) | 0.001 | 2.07 (1.34-3.21) | 0.001 |
| Any repeat revascularization | 66 (9.6) | 77 (4.7) | <.001 | 2.03 (1.46-2.82) | <.001 |
| Stent thrombosis | 1 (0.1) | 3 (0.2) | >.999 | 0.78 (0.08-7.49) | 0.829 |
| Patient-oriented composite endpoint | 74 (10.7) | 157 (9.6) | 0.438 | 1.12 (0.85-1.48) | 0.420 |
| Bleeding | 19 (2.8) | 53 (3.2) | 0.632 | 0.84 (0.50-1.42) | 0.514 |

**Supplementary Table 8.** Clinical outcome of patients with OCT analysis and those with angiography follow-up only.

|  | **OCT**  **(N=61)** | **Non-OCT**  **(N=628)** | ***P* (Chi-squared test)** | **Hazard ratio (95% confidence interval)** | | | ***P*** |
| --- | --- | --- | --- | --- | --- | --- | --- |
| Target lesion failure | 0 (0.0) | 28 (4.5) | 0.163 | | - | - | |
| Death |  |  |  | |  |  | |
| All-cause death | 0 (0.0) | 9 (1.4) | >.999 | | - | - | |
| Cardiac death | 0 (0.0) | 6 (1.0) | >.999 | | - | - | |
| Noncardiac death | 0 (0.0) | 3 (0.5) | >.999 | | - | - | |
| Target vessel-related myocardial infarction | 0 (0.0) | 3 (0.5) | >.999 | | - | - | |
| Any myocardial infarction | 0 (0.0) | 9 (1.4) | >.999 | | - | - | |
| Ischemia-driven target lesion revascularization | 0 (0.0) | 22 (3.5) | 0.247 | | - | - | |
| Ischemia-driven target vessel revascularization | 0 (0.0) | 38 (6.1) | 0.040 | | - | - | |
| Any repeat revascularization | 4 (6.6) | 62 (9.9) | 0.540 | | 0.64 (0.23-1.77) | 0.393 | |
| Stent thrombosis | 0 (0.0) | 1 (0.2) | >.999 | |  |  | |
| Patient-oriented composite endpoint | 4 (6.6) | 70 (11.1) | 0.374 | | 0.57 (0.21-1.56) | 0.374 | |
| Bleeding | 1 (1.6) | 18 (2.9) | >.999 | | 0.57 (0.08-4.24) | 0.579 | |
